# Supplementary material for: Dopamine neurons learn relative chosen value from probabilistic rewards
Source: eLife. 2016 Oct 27;5:e18044. doi: 10.7554/eLife.18044 (PMC5116238; doi:10.7554/eLife.18044)
Supplement: Supplementary file 1. — DOI: http://dx.doi.org/10.7554/eLife.18044.017 [file elife-18044-supp1.docx]

RW: Constant learning rate, no novelty term

RW&N: Constant learning rate, with novelty term

Decay: Decaying learning rate ($1/{t^{\kappa}}$), no novelty term

Decay&N: Decaying learning rate ($1/{t^{\kappa}}$),), with novelty term

PH: Adaptive learning rate ($\eta\left| PE \right|+(1-\eta)\alpha_{t-1}$), no novelty term

PH&N: Adaptive learning rate ($\eta\left| PE \right|+(1-\eta)\alpha_{t-1}$), with novelty term

| Model | $\boldsymbol{\alpha}$ | $\boldsymbol{\kappa}$ | $\boldsymbol{\eta}$ | $\boldsymbol{\alpha}_{\boldsymbol{1}}$ | $\boldsymbol{\tau}$ | BIC |
| --- | --- | --- | --- | --- | --- | --- |
| RW | 0.32 | NA | NA | NA | NA | 5990 |
| RW&N | 0.28 | NA | Na | NA | 1.03 | 5926 |
| Decay | NA | 1.32 | NA | NA | NA | 5948 |
| Decay&N | NA | 1.21 | NA | NA | 0.9 | 5913 |
| PH | NA | NA | 0.13 | 0.7 | NA | 5888 |
| PH&N | NA | NA | 0.13 | 0.7 | 0.95 | 5861 |

$\boldsymbol{\alpha}$: Fixed learning rate for novel cue

$\boldsymbol{\kappa}$: Decay constant for novel cue

$\boldsymbol{\eta}$ : Parameter of Pearce-Hall model for novel cue

$\boldsymbol{\alpha}_{\boldsymbol{1}}$: Learning rate on the first trial of Pearce-Hall model for novel cue

$\boldsymbol{\tau}$ : Decay constant of novelty term
