# Supplementary material for: Dopamine neurons learn relative chosen value from probabilistic rewards
Source: eLife. 2016 Oct 27;5:e18044. doi: 10.7554/eLife.18044 (PMC5116238; doi:10.7554/eLife.18044)
Supplement: Supplementary file 2. — DOI: http://dx.doi.org/10.7554/eLife.18044.018 [file elife-18044-supp2.docx]

RW: Constant learning rate, no novelty term

RW&N: Constant learning rate, with novelty term

Decay: Decaying learning rate ($1/{t^{\kappa}}$), no novelty term

Decay&N: Decaying learning rate ($1/{t^{\kappa}}$),), with novelty term

PH: Adaptive learning rate ($\eta\left| PE \right|+(1-\eta)\alpha_{t-1}$), no novelty term

PH&N: Adaptive learning rate ($\eta\left| PE \right|+(1-\eta)\alpha_{t-1}$), with novelty term

Monkey A

| Model | $\boldsymbol{\alpha}_{\boldsymbol{N}}$ | $\boldsymbol{\alpha}_{\boldsymbol{F}}$ | $\boldsymbol{\kappa}_{\boldsymbol{N}}$ | $\boldsymbol{\kappa}_{\boldsymbol{F}}$ | $\boldsymbol{\eta}_{\boldsymbol{N}}$ | $\boldsymbol{\eta}_{\boldsymbol{F}}$ | $\boldsymbol{\alpha}_{\boldsymbol{N}}\boldsymbol{(}\boldsymbol{1}\boldsymbol{)}$ | $\boldsymbol{\alpha}_{\boldsymbol{F}}\boldsymbol{(}\boldsymbol{1}\boldsymbol{)}$ | $\boldsymbol{\tau}$ | $\boldsymbol{\beta}$ | BIC |
| --- | --- | --- | --- | --- | --- | --- | --- | --- | --- | --- | --- |
| RW | 0.4 | 0.13 | NA | NA | NA | NA | NA | NA | NA | 0.09 | 2833 |
| RW&N | 0.4 | 0.11 | NA | NA | NA | NA | NA | NA | 1.21 | 0.07 | 2817 |
| Decay | NA | NA | 0.5 | 0.89 | NA | NA | NA | NA | NA | 0.09 | 2831 |
| Decay&N | NA | NA | 0.54 | 0.94 | NA | NA | NA | NA | 0.47 | 0.09 | 2823 |
| PH | NA | NA | NA | NA | 0.31 | 0.22 | 0.67 | 0.06 | NA | 0.09 | 2812 |
| PH&N | NA | NA | NA | NA | 0.25 | 0.15 | 0.7 | 0.05 | 0.91 | 0.08 | 2682 |

Monkey B

| Model | $\boldsymbol{\alpha}_{\boldsymbol{N}}$ | $\boldsymbol{\alpha}_{\boldsymbol{F}}$ | $\boldsymbol{\kappa}_{\boldsymbol{N}}$ | $\boldsymbol{\kappa}_{\boldsymbol{F}}$ | $\boldsymbol{\eta}_{\boldsymbol{N}}$ | $\boldsymbol{\eta}_{\boldsymbol{F}}$ | $\boldsymbol{\alpha}_{\boldsymbol{N}}\boldsymbol{(}\boldsymbol{1}\boldsymbol{)}$ | $\boldsymbol{\alpha}_{\boldsymbol{F}}\boldsymbol{(}\boldsymbol{1}\boldsymbol{)}$ | $\boldsymbol{\tau}$ | $\boldsymbol{\beta}$ | BIC |
| --- | --- | --- | --- | --- | --- | --- | --- | --- | --- | --- | --- |
| RW | 0.39 | 0.15 | NA | NA | NA | NA | NA | NA | NA | 0.08 | 1718 |
| RW&N | 0.39 | 0.16 | NA | NA | NA | NA | NA | NA | 1.23 | 0.08 | 1697 |
| Decay | NA | NA | 0.49 | 0.83 | NA | NA | NA | NA | NA | 0.08 | 1689 |
| Decay&N | NA | NA | 0.36 | 0.72 | NA | NA | NA | NA | 0.81 | 0.07 | 1688 |
| PH | NA | NA | NA | NA | 0.28 | 0.19 | 0.66 | 0.07 | NA | 0.08 | 1685 |
| PH&N | NA | NA | NA | NA | 0.23 | 0.14 | 0.7 | 0.04 | 0.99 | 0.08 | 1621 |

$\alpha_{N}$ : Fixed learning rate for novel cue

$\alpha_{F}$ : Fixed learning rate for familiar cue

$\kappa_{N}$ : Decay constant for novel cue

$\kappa_{F}$ : Decay constant for familiar cue

$\eta_{N}$ : Parameter of Pearce-Hall model for novel cue

$\eta_{F}$ : Parameter of Pearce-Hall model for familiar cue

$\alpha_{N}(1)$: Learning rate on the first trial of Pearce-Hall model for novel cue

$\alpha_{F}(1)$: Learning rate on the first trial of Pearce-Hall model for familiar cue

$\tau$ : Decay constant of novelty term

$\beta$ : Temperature parameter of softmax function
